# An EigenFactor-weighted power mean generalization of the Euclidean Index

**DOI:** 10.1371/journal.pone.0212760

**Published:** 2019-02-22

**Authors:** M. Ryan Haley

**Affiliations:** Department of Economics, University of Wisconsin Oshkosh, Oshkosh, WI, United States of America; University of Pittsburgh, UNITED STATES

## Abstract

This paper proposes a weighted generalization of the recently developed Euclidean Index. The weighting mechanism is designed to reflect the reputation of the journal within which an article appears. The weights are constructed using the Eigenfactor Article Influence percentiles scores. The rationale for assigning weights is that citations in more prestigious journals should be adjusted to logically reflect higher costs of production and higher vetting standards, and to partially counter several pragmatic issues surrounding truncated citation counts. Simulated and empirical demonstrations of the proposed approaches are included, which emphasize the flexibility and efficacy of the proposed generalization.

## Introduction

Assessing scholarly output, such as research articles, is an inescapable aspect of academia. Renewal, promotion, tenure, professorial awards, etc. invariably require an assessment of an applicant’s scholarly accomplishments. How best to complete this task is a perennial debate, often sparking impassioned opinions spanning a broad array of subjective and objective considerations. In an ideal world, unbiased experts would have ample time to assess each case. However, the reality is that many who perform assessment (e.g., administrators) are neither unbiased nor have the requisite time or knowledge to consistently make sound judgments. Methods based on citation counts typically offer a degree of objectivity that may help mitigate subjective biases; however, these approaches also have shortcomings and implementational nuances.

Broadly speaking, the ranking of scholarly output, journal articles being the principle focus herein, has two primary forms. The first are *stated preference methods*, which revolve around expert opinion, whether gathered by survey or through first-hand review of materials (where feasible). The second primary school of thought revolves around *revealed preference methods*, which are typically based on citation counts in various ways. The revealed preference concept weighs how an article plays though the academic network; as an article moves into publication, the citations it earns are thought to reflect (i.e., reveal) the degree to which the literature values the article. Hybrids also exist; e.g., a revealed preference mechanism might be parameterized using an expert survey; [1] proceeds in such a manner.

Bibliometricians spend a great deal of time and effort creating and testing scholar assessment mechanisms, particularly revealed preference methods. Advances often add value by proffering simple and intuitive scholar-ranking rules that are grounded in agreeable axioms. A recent revealed-preference-based effort in this spirit is Perry and Reny’s Euclidean Index [2]. In their paradigm, any citation list is a member of the set *L*, defined as the set of all non-increasing sequences of non-negative real numbers. They define a *citation index* as any continuous function ι:L→R.

A primary and ongoing discussion in revealed-preference Bibliometrics concerns the function *ι*, which converts citation lists into some measure of citation impact. Many candidates have been posed [3], but many are rules-of-thumb without firm grounding in thoughtful axioms. While not the first (see, for example, [4] or [5]), Perry and Reny set forth five axioms (pg. 2725-2727) designed to guide the *ι*-selection process: Monotonicity, Independence, Depth Relevance, Scale Invariance, and Directional Consistency. Axioms (*i*), (*ii*), and (*iv*) isolate a family of index functions that are proportional to the generalized mean function:

$$\iota \left(x;\sigma \right)={\left(\sum_{i=1}^{n}x_{i}^{\sigma }\right)}^{\frac{1}{\sigma }},$$ (1)

where **x** ≡ (*x*_1_, …, *x*_*n*_) ∈ L is a scholar’s ranked citation list, *σ* > 0, and *n* indicates the number of articles. Axiom (*iii*) —Depth Relevance—restricts *σ* to be greater than one, and axiom (*v*) isolates the *σ* = 2 case; i.e., Perry and Reny’s *Euclidean Index*:

$$\iota_{E}\left(x\right)=\sqrt{\sum_{i=1}^{n}x_{i}^{2}}.$$ (2)

Thus, the newly proposed ordinal index simply computes the Euclidean length of a scholar’s citation list, which permits comparisons across scholars in the same field, time frame, career level, etc. Here is a simple example: Suppose a scholar has three articles with 30, 18, and 8 citations, respectively. This scholar’s “citation list” is written as **x** = [30, 18, 8]; i.e., the citation counts listed in descending order. The Euclidean Index takes this list and computes an index value that summarizes this scholar’s “citational” achievement with a single number. Specifically,

$$\iota_{E}\left(\left[30,18,8\right]\right)=\sqrt{30^{2}+18^{2}+9^{2}}=36.12$$

This process is then repeated for all scholars that one seeks to compare, and then their scores can be compared.

While *ι*_*E*_(**x**) has many attractive features, such as simplicity and axiom compliance, it has received some criticism as well. Articulating and expanding upon these shortcomings, and then proposing solutions to these shortcomings, is the general focus of this paper.

Regarding the shortcomings: Ng [1] and Andersen [6] investigated *ι*_*E*_(**x**) in some detail, and offer several key insights into the general contribution and performance of the newly minted Euclidean Index. Ng takes issue with axiom (*v*), Directional Consistency, questioning its “compellingness.” Ng also explores axiom (*iii*) —Depth Relevance —by correctly noting that the “depth” of the depth relevance is controlled by the *σ* value depicted in Eq (1). Perry and Reny (pg. 2726) describe depth relevance as follows:

It is often suggested that a good index should encourage “quality over quantity”, i.e., encourage a smaller number of highly cited papers over a larger number of infrequently cited papers… [Depth Relevance] says that it should not be the case that for any fixed number of citations, the index is maximized by spreading them as thinly as possible across as many publications as possible.

Ng goes on to suggest, based on a small-scale survey of academics, that 1 < *σ* < 2 may be preferred in practice over the *σ* = 2 value espoused by Perry and Reny; Ng specifically notes that *σ* = 1.6 was the most preferred value in the survey. A value of *σ* = 1.6 will still reward citations concentrated in a smaller number of papers, but does so less aggressively than does the *σ* = 2 value (the Euclidean Index).

Andersen’s critique is more technical, focusing on the level of redundancy exhibited by *ι*_*E*_(**x**) relative to several standard and/or closely related metrics. He essentially determines that *ι*_*E*_(**x**) offers little new information, meaning that *ι*_*E*_(**x**) scholar rankings are highly correlated with existing methods. He does note, however, that *ι*_*E*_(**x**) offers respectable stability intervals, which is encouraging. Andersen also emphasizes how *ι*_*E*_(**x**), with its squared function, exacerbates the Matthew Effect to an arguably distasteful degree; specifically, *ι*_*E*_(**x**) increases more aggressively if a new citation is added to a highly cited article as opposed to the new citation being added to a lesser cited article. Andersen also criticizes the units of *ι*_*E*_(**x**), Depth Relevance (axiom (*iii*)), the general focus on ranking scholars, and how the index can lead to dramatically different *ι*_*E*_(**x**) for scholars with the same number of total citations.

At the intersection of Ng and Andersen is a general belief, and supporting evidence, that the *σ* = 2 parameterization is too extreme, resulting in unrealistic and undesirable index properties. The purpose of this paper is to expose another shortcoming of the Euclidean Index, namely its failure to accommodate journal reputation differentials when assessing citations. This leads to the reverse problem noted by Andersen (pg. 462); i.e., two scholars with the same total citation counts can have the same *ι*_*E*_(**x**) value even if the first scholar has published all their articles in an elite journal while the other scholar has published all their articles in a very low-level journal. The final aim is to propose a weighted generalization of the Euclidean Index that at least partially relieves some of the issues raised by Ng and Andersen, but in a way that leverages the best of the Perry-Reny axioms and the generalized mean function.

Hereafter in this article, the discussion will observe the following conditions when assessing a citation list:

**Condition 1** *Each scholar’s citations should be reported on a per-author basis; e.g., if an article has Y citations and three authors, each author should be attributed Y*/3 *citations; equivalent approaches would also suffice.For example*, [7] *suggests a partial-authoring approach that might be adapted to this purpose*.
**Condition 2** *In circumstances where publication dates differ significantly, citations accumulated by each article should be divided by the number of years since publication to account for article age; equivalent methods that also adjust for academic age would also suffice. See, for example*, [8], [9], [10], *or* [11].
**Condition 3** *All scholars are presumed to be in the same field and publishing in the same sphere of academic journals, or have otherwise had their citation lists adjusted to permit inter-field comparisons. See, for example*, [12], [13], [14], [15], [16], *or* [17].
**Condition 4** *All citations lists should be equally treated insofar as self citations. See, for example*, [18].

Without these conditions, confusion can arise in the scholar comparison process. For example, comparing scholars from different fields is often not “apples-to-apples” due to differences in field size, journal count, citation culture, among other confounders. While not a complete list, adhering to these four conditions can help ensure comparisons are made where they are most likely to be reasonable.

The remainder of the paper is separated into three sections. Section two collects additional background information germane to the development of the *Weighted Euclidean Index* and *Weighted Power Mean Index*, which are proposed, discussed, and demonstrated in section three. Discussion, limitations, and conclusions appear in section four.

## Citations and journal reputation

Despite its attractive attributes, *ι*_*E*_(**x**) does not consider the reputation of journal within which an article appears, an omission that can precipitate peculiar results when applied. To demonstrate, consider Scholar 1 and Scholar 2 in Table 1. Clearly, both scholars have the same *ι*_*E*_(**x**) score equal to 138.29, yet Scholar 1 would likely be judged more accomplished by most well-informed impartial spectators (in the author’s field, at least). As a second example, consider comparing Scholars 2 and 3, the latter of which has a considerably lower *ι*_*E*_(**x**) score despite publishing all four articles in elite journals. In this case, too, one wonders if ranking Scholar 3 lower than Scholar 2 is sound.

**Table 1:** Scholar Comparison Example^†^.

Article	Scholar 1	Scholar 1	Scholar 2	Scholar 2	Scholar 3	Scholar 3
Rank	Citations	Journals	Citations	Journals	Citations	Journals
1	120	Elite	120	Fair	40	Elite
2	60	Elite	60	Fair	30	Elite
3	30	Elite	30	Fair	20	Elite
4	15	Elite	15	Fair	10	Elite
*ι*_*E*_(**x**)	138.29		138.29		54.77	

^†^All three scholars are assumed to have four articles, each of which has accumulated the listed number of citations. Journal reputation is also included in a generic fashion, marked as either “Elite” or “Fair.”

As a third example, consider the tenure cases of two junior scholars, each with four recently published articles; see Table 2. Scholar A has four articles, all in elite journals, whereas scholar B has published exclusively in much lower-level journals. Interpreting this directly would imply that Scholar A is inferior to Scholar B, which is hard to comprehend. This un-intuitive assessment result is not unique to *ι*_*E*_(**x**), but it does emphasize that even the newest, axiom-based metrics can have nontrivial shortcomings in practical settings (which is the implied audience in Perry and Reny).

**Table 2:** Junior Scholar Comparison Example^†^.

Article	Scholar A	Scholar A	Scholar B	Scholar B
Rank	Citations	Journals	Citations	Journals
1	4	Elite	7	Fair
2	3	Elite	4	Fair
3	2	Elite	2	Fair
4	1	Elite	1	Fair
*ι*_*E*_(**x**)	5.48		8.37	

^†^Both scholars are assumed to have four recently published articles when facing tenure. Journal reputation is also included in a generic fashion, marked as either “Elite” or “Fair.”

The primary point raised in Tables 1 and 2 is that ignoring the reputation of a journal within which an article appears can lead to unpalatable scholar rankings. But why should journal reputation, howsoever measured, be incorporated into a scholar’s research assessment?’ Five arguments for doing so appear below.

**Argument 1** *One argument for including journal reputation in the scholar assessment process is that articles published in elite journals are often especially hard earned, requiring many months and possibly even years of effort for the creative process to move from idea, to refined idea, to narrative, to seminar, to seminar again and again, to reviewed narrative, to edited narrative, to re-reviewed narrative, to re-edited narrative, and so on, until final and not-a-foregone-conclusion acceptance. In this vein, we are thinking in the spirit of what economists call the Smithian “labor theory of value” or the related Ricardian “cost of production theory of value”, both of which imply, once adapted to this context, that successfully publishing in an elite journal may warrant some extra accommodation. Referring again to* Table 1, *it is plausible to conclude that Scholar 1 exerted far more research effort (i.e., time, skill, creativity) than Scholar 2; it is plausible to conclude the same of Scholar A in* Table 2.
**Argument 2** *A second argument for including journal reputation in the scholar assessment process concerns the phrase “article quality.” Is it reasonable to conclude than an article is “high quality” if and only if it has a high citation count? Quality surely has other germane meanings in academics. For example, quality might instead mean that the article is unusually creative (perhaps even landmark); technically sophisticated and current (e.g., leading edge inference techniques were included and executed properly); especially complete (e.g., of broad enough scale to assuage small-sample concerns and other fragilities); thoroughly vetted by top-level peers; among any number of additional virtues we might expect from a paper labelled “high quality.” Importantly, citation count does not necessarily guarantee any of these merits. In contrast, journal quality can more directly ensure that these virtues are realized, as these features will generally be necessary to successfully navigate through the gate-keepers at elite journals, which themselves have a strong incentive to uphold the reputation of the journal. Moreover, it is quixotic to conclude the opposite; i.e., that lesser journals have higher standards in these regards*.
**Argument 3** *A third argument for including journal reputation in the scholar assessment process is that, for good or ill, journal reputation is likely to be of preeminent importance to junior faculty because tenure clocks are quite short and often do not permit enough time for citation counts to accumulate. Indeed, tenure is often decided using very “young” articles, some of which will be forthcoming or perhaps still in the revise and resubmit phase when evaluated. Relatedly, a more senior scholar seeking a new, more esteemed position may well be judged based on the reputation of the journals housing her more recent publications, which have likewise had minimal time to accumulate citations*.
**Argument 4** *A fourth argument for including journal reputation in the scholar assessment process comes from scholars themselves. For example*, [19] *find “…holding other things constant, adding publications in lower-rated journals to what is typically considered a good publication record does have a significant negative impact on economists’ judgments of the value of the author’s contribution.” This study is based on economists specifically, but citations therein point to similar less-is-more, better-is-better mentalities in other fields as well (e.g., Psychology)*.
**Argument 5** *A fifth argument for including journal reputation in the scholar assessment process stems from Nobel Laureate Michael Spence’s (1973) Signalling Theory*, [20]. *His paradigm describes how costly educational attainment serves as a method for agents (workers) to signal their abilities to principals (firms) in the presence of asymmetric (unobservable) information about worker ability. Because the signal is more costly for low-ability worker types, the firm can infer a positive correlation between educational attainment and ability, which in turn helps them form salary offers. This adapts well to this context; specifically, journal reputation can serve as useful signalling information in the hiring, promoting, tenuring, and awarding contexts often found in higher education*.

In contrast to these arguments, some scholars reject the notion of using journal reputation measures (e.g., Impact Factors) to gauge article quality (e.g., [21]; DORA). Oswald ([22]) demonstrates a similar point by noting that over a 25-year span, it is better to have published a best article (in a citation count sense) in the journal *Oxford Bulletin of Economics and Statistics* (OBES) (a solid “A-” journal in Economics) than to have published all four of the worst articles in *The American Economic Review* (AER), arguably the most prestigious journal in Economics. He argues that decision-makers (e.g., promotion/tenure committees, award committees) need to understand the implication, which is that the prowess of the journal within which an article appears should not necessarily be used to judge the quality of an article. In his words:

This paper is a simple one. It provides evidence that it is dangerous to believe that a publication in a famous journal X is more important than one published in medium-quality journal Y… the publication system routinely pushes high-quality papers into medium-quality journals, and vice versa.”

While Oswald’s critique surely has merit, perhaps it is equally dangerous to over-correct and presume that a typical acceptance at the AER is equivalent to a typical acceptance at OBES. In fact, one might contend that it is an even *greater* feat to publish a marginal paper in the AER than to publish an elite-quality article in OBES. Moreover, if journal reputation does not matter and the same citation count can be achieved in OBES, then why would any scholar aspire to the AER? Why not simply send all A and A- level articles to OBES, and thereby enjoy an expedited and less onerous review process with less anxiety about possible rejection? That we empirically observe great interest in publishing in an elite journal like the AER suggests there is more afoot than citation counts ([23]). At minimum, it would seem, success at the AER signals (in the Spencean sense) a level of creativity, work ethic, and/or human capital that is not shared by all economists. Should the journal labels really be inconsequential? Perhaps not. In fact, Oswald himself is not entirely dismissive of this point: “… [journal] reputation ratings in academia have their uses, and it is unlikely that any scholar would argue that labels are meaningless.” It is perhaps also worth noting that Oswald is careful to frame his argument around elite vs. very good journals; he does not explicitly address the case where the lesser journal is a very low-level journal.

With these arguments, the counter arguments, and the critiques from [1]) and [6] withstanding, the goal hereafter is to deliver a scholar-ranking method with two core properties:

1. A citation weighting mechanism that at least partially resolves the shortcoming apparent in Tables 1 and 2 by incorporating journal reputation into the citation-assessment process.
2. A flexible way to moderate the degree of depth relevance. This addresses Ng’s primary concern and also at least reduces the Matthew Effect noted by Andersen.

## A weighted generalization of the Euclidean index

Perhaps the simplest way to achieve the two desired properties noted in the prior section is to a) weight an article’s citations to reflect the reputation of the journal within which the article appears and b) form a generalized version of the Euclidean Index wherein the *σ* value can range more widely than the *σ* = 2 value imposed by axiom (*v*) from Perry and Reny. Each of these goals is addressed in a subsection below.

### Citation weighting

The weighting mechanism should be a positive monotonic function of a credible measure of journal reputation. Convenient and established ways to assess journal reputation include the Eigenfactor (*EFp*) and Article Influence (*AIp*) percentile scores from eigenfactor.org; see, for example, [24], [25], [26], or [27]. These metrics measure a journal’s prowess within its field using a percentile mechanism. That the *EFp* and *AIp* are contained on the unit interval makes them especially convenient choices for weighting. Hereafter, the focus will be on *AIp* for reasons explained below, though the analysis could, of course, be easily paralleled using the *EFp*, if so desired.

The Eigenfactor metric is a network-based measure of citation accumulation. Perhaps the most eloquent description of how it works appears in [24] (pg. 238):

Imagine that a researcher is to spend all eternity in the library randomly following citations within scientific periodicals. The researcher begins by picking a random journal in the library. From this volume she selects a random citation. She then walks over to the journal referenced by this citation. From this new volume she now selects another random citation and proceeds to that journal. This process is repeated ad infinitum.

So when we report that [the journal] Nature had an Eigenfactor score of 2.0 in 2006, that means that two percent of the time, the model researcher would have been directed to Nature.

[24] (pg. 239) also define the Article Influence Score as follows:

The Article Influence Score is calculated as a journal’s Eigenfactor Score divided by the number of articles in that journal, normalized so that the average article in the Journal Citation Reports has an Article Influence Score of 1.

In short, the Eigenfactor index determines journal reputation using a journal’s actual “popularity” (measured in citation counts) within the corresponding network of academic journals. This network is very large, and includes all journals listed in the Thomson-Reuters Journal Citation Report, which contains thousands of journals. The *AIp* score reports the *AI* score of each journal as a percentile within the journal’s ISI category. For example, the aforementioned AER and OBES belong to the Economics ISI category, and have *AIp* scores of 99% and 79%, respectively. These values can be interpreted like a standard percentile; e.g., OBES has an *AI* value higher than 79% of the other journals in the Economics ISI category. These percentiles are a measure of a journal’s reputation, and as such they adapt well to the weighting process requirements; e.g., the citation count of an article in OBES is scaled by 0.79 to reflect not only the citation count, but also the reputation (measured as a percentile within the field) of the journal within which the article is published.

While other weighting choices surely exist, such as Impact Factors or *h*-index values, both would require some extensive modifications before being suitable as weights. For example, the *h*-indices for all journals within a specific field would have to be mapped to the [0, 1] interval before they would meet the mathematical definition of a proper weight; Impact Factors would require a similar conversion. While this may be possible, it is not trivial. In contrast, the Eigenfactor percentiles measures are directly available from www.eigenfactor.org at no cost and require no transformations prior to being used as weights. Additionally, the *AI* value is very similar to the Impact Factor insofar as it adjusts for journal size. For these reasons, the *AIp* is the focus hereafter. (This is not the first attempt to create an author-level metrics using the eigenfactor metrics; see, for example, [25] and [26]).

Combining the Euclidean Index with the *AIp* weighting scheme produces the *Weighted Euclidean Index*:

$$\iota_{W}\left(x;\sigma =2\right)\equiv {\left[\sum_{i=1}^{n}AIp_{i}x_{i}^{2}\right]}^{\frac{1}{2}},$$ (3)

where *w*_*i*_ ≡ *AIp*_*i*_. Note that the weighted citations are also elements of the set *L* and that *ι*_*W*_(**x**, *σ* = 2) ≤ *ι*_*E*_(**x**) ∀ **x** ∈ *L*. Before demonstrating this approach, a generalization is proposed in the following subsection.

### The weighted power mean index

To achieve the second property, namely less severe values for *σ*, and to correspondingly ease concerns about the Matthew Effect and Depth Relevance, consider a generalized version of Eq (3):

$$\iota_{W}\left(x;\sigma \right)\equiv {\left[\sum_{i=1}^{n}AIp_{i}x_{i}^{\sigma }\right]}^{\frac{1}{\sigma }},$$ (4)

where *σ* controls the degree of depth relevance. Depth relevance can be thought of as the degree to which outlier citation counts are valued; the higher the *σ* value, the more a “one-hit-wonder” article impacts the index score. Eq (4) is hereafter called the *Weighted Power Mean Index*, which reduces to the Weighted Euclidean Index in the special case when *σ* = 2.

To demonstrate *ι*_*W*_(**x**; *σ*), consider Table 3, which contains the same simulated data from Table 1 except journal reputation is now explicitly measured using *AIp* values. The index scores are shown for four different *σ* values from the [1, 2] interval.

**Table 3:** **Scholar Comparison Example:** With weighting^†^.

Article	Scholar 1	Scholar 1	Scholar 2	Scholar 2	Scholar 3	Scholar 3
Rank	Citations	Journal *AIp*	Citations	Journal *AIp*	Citations	Journal *AIp*
1	120	0.99	120	0.37	40	0.99
2	60	0.97	60	0.32	30	0.97
3	30	0.96	30	0.24	20	0.96
4	15	0.91	15	0.11	10	0.91
*ι*_*W*_(**x**; *σ* = 2)		137.17		81.98		54.15
*ι*_*W*_(**x**; *σ* = 1.6)		151.18		78.36		62.03
*ι*_*W*_(**x**; *σ* = 1.2)		183.91		74.44		79.12
*ι*_*W*_(**x**; *σ* = 1.0)		219.45		72.45		97.00

^†^When interpreting the index values, note that, like the original (unweighted) Euclidean Index, the weighted version is an ordinal measure; i.e., no cardinal properties are presumed. Note also that the *AIp* weights are in the unit interval, but need not necessarily sum to one. A weighted citation index scores is computed, for Scholar 1 for example, as follows:
$$\begin{array}{c} \iota_{W}\left(x;\sigma =1.6\right)={\left(0.99\times 120^{1.6}+0.97\times 60^{1.6}+0.96\times 30^{1.6}+0.91\times 15^{1.6}\right)}^{\frac{1}{1.6}}. \end{array}$$

Table 3 highlights several issues. First, when using *ι*_*W*_(**x**; *σ* = 2) the scholars are sorted in a more intuitively appealing manner than when *ι*_*E*_(**x**) is used. Most notably Scholars 1 and 2 no longer have identical scores, as they did in Table 1. Second, as per Andersen (2017), the Matthew Effect is most prominent for the *σ* = 2 case, which is controlled, or at least eased, by using lower values for *σ*. To see this, note how the outlier effect for Scholar 2’s first article is largely eroded by the *σ* = 1.2 value, wherein Scholar 3 overtakes Scholar 2. Table 4 contains a parallel analysis for the junior faculty example from Table 2; similarly improved conclusions are apparent.

**Table 4:** **Scholar Comparison Example:** Junior faculty with weighting.

Article	Scholar A	Scholar A	Scholar B	Scholar B
Rank	Citations	Journal *AIp*	Citations	Journal *AIp*
1	4	0.99	7	0.37
2	3	0.97	4	0.32
3	2	0.96	2	0.24
4	1	0.91	1	0.11
*ι*_*W*_(**x**; *σ* = 1.6)		4.09		1.69

Those that believe that one citation should be counted as one citation, irrespective of the journal that published the article, may be disinclined to acquiesce to the weighted scholar rankings in Tables 3 and 4. However, those that see value in intertwining citations with journal reputation may find the comparisons more suitable than comparing unweighted *ι*_*E*_(**x**) values. Put another way, both versions are imperfect, but the chance of grossly mis-ranking a scholar is perhaps more likely when journal reputation is entirely ignored.

### Empirical demonstration

To demonstrate the potential value of the weighting approach, Google Scholar citation data for 10 experienced (associate level+) micro-economists from the University of Wisconsin System was collected for the 2014-2017 time frame; articles published outside this window were excluded. These scholars were randomly selected from the three largest campuses, which have different levels of research expectations, denoted as either R1 or R2. More specifically, the flagship campus is unequivocally an R1, whereas the other two campuses were recently R2 or are aspiring to become R2. Working papers (e.g., Research Gate, SSRN, etc.) were excluded, as were books. This is not to that imply working papers and books are not valuable scholarly efforts, but rather to focus the assessment on published journal articles specifically. The data are de-identified, but are otherwise accurate as of October 2018. A summary of the data, and concomitant results, appear in Table 5.

**Table 5:** Empirical scholar comparison^†^.

ID	Scholar	Total Citations /Author/Year	Article Count	*ι*_*W*_(**x**; *σ* = 2)	*ι*_*W*_(**x**; *σ* = 1.6)	*ι*_*W*_(**x**)
R2	1	31.00	11	9.50	10.14	15.99
R2	2	18.58	15	5.56	6.88	6.12
R2	3	2.69	3	1.85	7.12	2.01
R2	4	32.5	9	7.48	8.18	13.98
R2	5	13.5	6	4.54	4.72	6.91
R2	6	31.58	22	7.69	9.88	8.66
R1	7	19.08	4	11.10	12.36	11.39
R1	8	39.17	6	21.41	24.21	21.44
R1	9	16.67	4	9.19	10.40	9.38
R1	10	26.08	2	21.07	21.92	21.07

^†^The data includes citation information for 10 tenured micro-economists from the three largest University of Wisconsin System campuses. All citation counts were obtained from Google Scholar in October 2018, and all journals were weighted using Eigenfactor *AIp* values; if an *AIp* value could not be obtained, which happened in a handful of instances, weights were imputed by cross referencing the journal’s nearest neighbors on the REPEC 10-year Simple Impact Factor list with *AIp* values for the nearest-neighbor journals. All citation counts were adjusted to align with Conditions 1-4.

The ten citations lists were subjected to Conditions 1-4 set forth earlier in the paper. Specifically, all article citations counts were divided by the number of authors, as per Condition 1; all citation counts were divided by the number of years since publication, as per Condition 2; all authors were from the same sub-field of Economics, as per Condition 3; and all citation lists included self citations, as per Condition 4.

In a handful of cases, *AIp* values could not be obtained because the journal was not available in the Eigenfactor database. In such instances, weights were imputed by cross referencing the journal’s nearest neighbors on the REPEC 10-year Simple Impact Factor List with *AIp* values for the nearest-neighbor journals.

One would reasonably expect scholars from the R1 campus to have higher index values than scholars from the R2 campuses. On this matter, consider first the total citations/author/year column in Table 5, which clearly reveals a pattern counter to this expectation; i.e., there are multiple R2 scholars ranked higher than multiple R1 scholars. This suggests that the unweighted *σ* = 1 case (i.e., the simple sum-of-citations) does not deliver intuitively appealing results.

Second, consider the *ι*_*E*_(**x**) scores (the rightmost column in Table 5), the results of which are more in line with expectations, but still not entirely satisfying; i.e., there are two scholars (scholars 1 and 4) from the R2 campuses that score higher than Scholars 7 and 9 from the R1 campus. In contrast, the Weighted Euclidean Index, *ι*_*W*_(**x**; *σ* = 2), delivers a scholar ranking more consistent with expectations, as does the Weighted Power Mean Index that uses the *σ* = 1.6 value suggested by Ng (2017). Specifically, the R1 scholars consistently score higher than the R2 scholars.

Another insight evinced by Table 5 concerns metric variations for certain scholars. Perhaps the most interesting cases are Scholars 1 and 4, both of whom have particularly high (unweighted) Euclidean Index scores (for an R2), but more modest Weighted Euclidean Index scores. A close examination of these scholars’ work reveals that many of their articles appear in journals with medium-to-low *AIp* scores, which are ignored by the standard (unweighted) Euclidean Index but accounted for by the Weighted Euclidean Index and Weighted Power Mean Index. Note that this pattern is reversed for the R1 scholars because they tend to publish in only high-level journals. Another interesting pattern concerns the number of articles, which is generally lower for the R1 scholars; despite this, however, they have higher Weighted Euclidean Index and Weighted Power Mean Index values, which reflects the fact that they tend to publish in, and often only in, the most elite journals.

## Discussion, limitations, and conclusions

This paper proposes a simple axiom-based way to build a practical scholar-ranking metric that incorporates the reputation of the journal in which an article appears. Tables 1 and 2 evince the need for adjustments of this type, and rhetorical arguments were also supplied to support incorporating journal reputation. The Weighted Euclidean Index and Weighted Power Mean Index are simple to apply and are based on established data. Importantly, the weights are based on *AIp* percentile journal rank scores, which are readily available, well vetted, and adapt easily to the weighting process. One subtle advantage of the weighting approach is that it can inhibit the effectiveness of scholar-level citation cartels ([28, 29]). When only citations matters, as with the standard Euclidean Index, only citations need to be gamed by the cartel; however, managing journal reputation is far more difficult for a scholar-level citation cartel.

As with any method, there are several limitations. First, to create the *AIp* weights, the corresponding journals must be listed in the Thomson-Reuters Journal Citation Report; while this source includes thousands of journals, it does not include all journals, and journal coverage varies by field. Second, all methods proposed herein, save for a brief mention of the sum-of-citations metric, employ some degree of depth relevance, which may or may not be ideal; the alternative, called *breadth relevance* —rewarding citations spread across many articles —may be preferred in selected settings. Third, because citation count data is at the core of the proposed methods, the database(s) from which they draw citation data must be complete and credible ([30]); this concern arises for all citation-based metrics, and those proposed herein are no exception. Fourth, any *σ* ∈ (1, 2) induces a metric that does not satisfy Perry and Reny’s fifth axiom; whether this is consequential remains a matter of opinion and future research; however, the fifth axiom is of dubious value (Ng, 2017) and thus bypassing it may be of little consequence. Fifth, this paper’s focus was on making a case for these modified metrics and demonstrating their value in a variety of simulated and small-scale empirical settings; the lack of a broad scale empirical study is accordingly a limitation, but one that could be re-mediated with future research. Additional directions for future research include extensions involving other possible weighting mechanisms, and comparisons thereof ([31]). Developing this possible approach could certainly leverage some of the concepts set forth in the present paper.

Finally, the primary goal of this work is to offer a simple and pragmatic mechanism to facilitate the comparison of scholarly accomplishments, principally research articles, in a way that jointly values citation counts and journal reputation. The ubiquitous caveat in Bibliometrics is that metrics, howsoever defined, should never be divorced from sound, vetted professional judgment; this caveat applies here as well. Indeed, it is somewhat disquieting to propose metrics for ranking scholars, but the alternative is to not and be subjected to potentially grievous mis-applications of poorly designed or poorly understood metrics. [25] offers two cogent advantages of scholar ranking, though they are careful to note that the ranking process can be easily abused by hurried administrators. [6] also stresses that ranking scholars is a delicate matter that can be difficult to recommend. Nonetheless, the ranking of scholars will persist, and too often said process will be implemented by officials with sub-par or biased assessment skills. Given this, it is perhaps more efficacious to offer simple, flexible, intuitively-appealing approaches that value both citation count and journal reputation. Perhaps the goal should be to create methods that minimize the maximum amount of damage decision-makers can do. Focusing only on citations leaves a lot of room for errors and focusing only on journal reputation also leaves room for errors; perhaps focusing on both offers an agreeable balance.
